# Treatment of Uterine Catarrh by Internal Application of Carbolic Acid

**Published:** 1870-08

**Authors:** W. Playfair

**Affiliations:** Physician to King’s College Hospital


### Treatment of Uterine Catarrh by Internal Application of Carbolic Acid.

By W. Playfair, M.D., Physician to King's College Hospital.

In a large proportion of old standing cases of uterine catarrh it is hopeless to effect a permanent cure by any means which do not act directly on the seat of the disease, which is the lining membrane of the cavity of the uterus and cervical canal beyond the external os, accompanied, of course, with secondary morbid states of the body of the uterus and cervix, such as hypertrophy, congestion, &c. Rest, applications to the exterior of the cervix, and general treatment will unquestionably cause a temporary improvement, but on a recurrence to the old habits of life all the old symptoms return. There are serious objections to intra-uterine injections, unless the os is first dilated with laminaria tents, as they are apt to bring on severe uterine colics. By means of fine probes of whalebone or flexible metal, round which a thin film of fine cotton-wool is wrapped, alterative applications can readily be made to the interior of the uterus, without pain or danger. In the very numerous cases in which this plan of treatment has been carried out, in no single instance has anything but the greatest benefit accrued. It is no doubt advisable to select the cases judiciously, and where there is much uterine tenderness, intra-uterine treatment should be postponed until this has been diminished by rest, leeching, &c.; but with proper precautions the treatment is perfectly safe. A concentrated solution of carbolic acid, eighty parts to twenty of water, is used; and it acts so well that for a long time nothing else has been employed. After the first application the discharge is sometimes increased, but after the second or third it is generally greatly diminished, and a single application is often sufficient to cure superficial erosions of the cervix. As a rule, there is no difficulty in passing the probes, as in true uterine catarrh the os is invariably patulous. As the case improves the patulous state of the os diminishes, and this is found to be one of the most certain signs of improvement.—*Medical News and Library.*

